# A functional trait database for Mediterranean Basin plants

**DOI:** 10.1038/sdata.2018.135

**Published:** 2018-07-10

**Authors:** Çağatay Tavşanoğlu, Juli G. Pausas

**Affiliations:** 1 Division of Ecology, Department of Biology, Hacettepe University, Beytepe 06800, Ankara, Turkey; 2 Centro de Investigaciones sobre Desertificación, Consejo Superior de Investigaciones Científicas (CIDE-CSIC), 46113, Valencia, Spain

**Keywords:** Plant ecology, Fire ecology, Community ecology

## Abstract

Functional trait databases are emerging as crucial tools for a wide range of ecological studies across the world. Here, we provide a database of functional traits for vascular plant species of the Mediterranean Basin. The database includes 25,764 individual records of 44 traits from 2,457 plant taxa distributed in 119 taxonomic families. This database (BROT 2.0) is an updated and enlarged version of a previous database (BROT 1.0; 8,263 records, 14 traits, 952 taxa). Trait data were obtained from a comprehensive literature review, plus some field and experimental observations. All records are fully referenced and, in many cases, include geographic coordinates. The database is structured to include different levels of accuracy of trait information for each entry. BROT 2.0 should facilitate testing hypotheses on plant functional ecology within the Mediterranean Basin, and comparing this region with other ecosystems worldwide. The BROT 2.0 database and its trait definitions can be used as a template for creating similar trait databases in other regions of the world.

## Background & Summary

Plant functional traits determine the response of plant species to environmental factors^1^ and disturbances^2^, and are currently used for testing a range of ecological hypotheses^3–6^. Trait-based approaches can also be used to understand the mechanisms assembling communities^7–10^. Exploring functional (trait) diversity in communities is providing new insights on ecological processes and ecosystem functioning^11,12^, and has a promising future in the ecology research agenda^13^. Plant trait databases are also important sources for evolutionary biologists; together with calibrated phylogenies, they are a source of information for understanding the origin and evolution of selective pressures^14–16^. Consequently, trait databases are becoming a key research tool in ecology and evolution.

The Mediterranean Basin harbors around 25 000 plant species, including many endemics^17^, and has a unique plant genetic diversity within the western Palearctic biogeographic region, mainly attributed to the role of the area as glacial refugium during Pleistocene glacial periods^18,19^. The presence of many peninsulas (Iberian, Italian, Balkan, and Anatolian) and islands is another driver of plant endemism in the region. Furthermore, the geographical position of the basin at the intersection of three continents (Europe, Asia, and Africa) has shaped a unique plant assembly with plants from the three different biogeographical regions^20^. At the same time, however, the Mediterranean Basin is also considered one of the global biodiversity hotspots for habitat loss^21^. This region is the cradle of Western civilization, and has witnessed the rise and fall of many cultures, some with very high population densities, that have led to high levels of habitat degradation and loss for many centuries. Recent threats to plant diversity in Mediterranean Basin include changes in climate, land use and fire regime^22,23^. Therefore, a description of functional trait structure and variability across the Mediterranean Basin is of critical importance for predicting the possible responses of plant populations, species and communities to future changes.

The creation of the BROT database^24^ (hereafter, BROT 1.0) was a major effort to gather plant functional traits for the Mediterranean region. One of the strengths of this database was that it included a range of data types for each trait (e.g., quantitative, semi-quantitative, and binary) so that even coarse information was stored in a useful way within the database, together with high-quality data. It also considered variability within species by including, when available, several trait records for a given taxon. The information included in the database came from many references, including gray literature (reports, theses, and articles in various languages) that are not easily accessible to users. BROT 1.0 was a first step in compiling and organizing trait information in a standardized format for international users. Although BROT 1.0 was focused on fire-related and regeneration traits^24^, it has been used in many papers published in peer-reviewed journals (more than 150 GoogleScholar citations by December 2017) and was also included in a global plant trait database (TRY^25^) and in the Encyclopedia of Life (eol.org).

The increase in functional trait research for the Mediterranean flora, together with the need for a more comprehensive plant trait database for studies on functional ecology and evolution, motivated us to make a major update of the BROT database. Here we introduce BROT 2.0. The great effort performed in compiling regeneration traits in BROT 1.0 was continued in this update, making BROT 2.0 a prominent database on regeneration traits for Mediterranean flora, and in particular for traits related to the regeneration after disturbance. In addition, in this new version of BROT we have increased (tripled) the number of traits and taxa, improved the standardization of some traits, and added geographical coordinates (where possible) for enabling geographically explicit analyses. This new version includes not only regeneration traits, but also vegetative and sexual reproductive traits. Thus BROT 2.0 is the largest source of information on plant traits for the Mediterranean Basin region.

**Figure 1:**
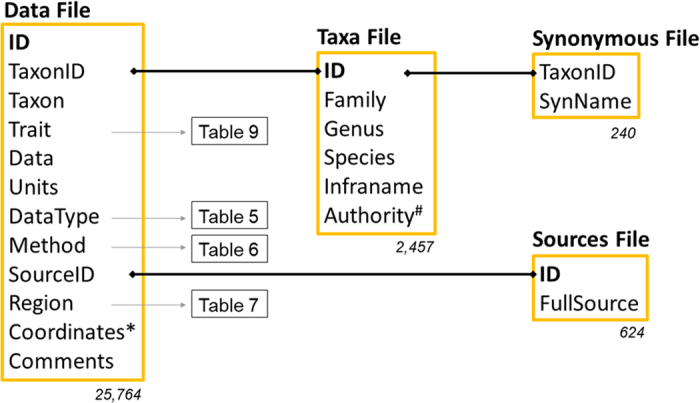
Structure of BROT 2.0 database. Yellow boxes are the four files that make up the database, and include the column names of each file and the number of rows (below the box); ID refers to unique identifiers in the corresponding file. Black thick lines indicate the link between files. Gray arrows indicate the manuscript table in which information on a column can be found. (*) “Coordinates” include four columns: latitude, longitude, altitude, and accuracy (see Table 1). (#) “Authority” includes two columns: the authority for the species binomial name, and for the infra-species category (see Table 2).

The BROT 2.0 database is structured on four files (Fig. 1), all connected by unique identifiers. The fact that most information is in the ‘Data file’, and in plain text, simplifies the use of the database. We propose BROT 2.0 as a reference database for functional trait analyses and hypothesis testing within the Mediterranean Basin and for comparing this area with other ecosystems across the globe. It is also a useful tool for identifying knowledge gaps on the functional ecology of plants in the study region. The database and its standardized definitions may also be used as a template for compiling plant trait databases in other regions of the world.

## Methods

### Database structure

The structure of BROT 2.0 is very simple and composed of four files (Fig. 1), all included in a single dataset (Data Citation 1). The ‘Data file’ includes the main data values for functional traits, the taxon name, and the geographical information associated with each record (Table 1). The ‘Taxa file’ consists of the full scientific name of all taxa, including the authority (Table 2). The ‘Sources file’ includes the full reference for each source used in the database (Table 3). An additional file includes synonymous names for the taxa considered in the database (Table 4). These four files are linked by unique identifiers (Fig. 1). Additional information on the Data file is given in Tables 5–8 (Fig. 1). The list of traits are provided in Table 9 and a full description and standardization of all traits are given in the ‘Data Records’ section.

### Data compilation

**Figure 2:**
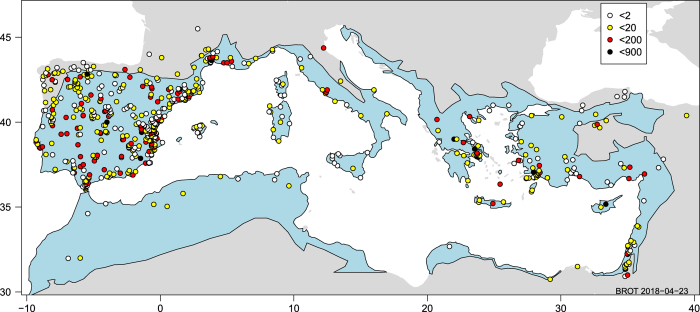
Geographical scope of the database in the Mediterranean Basin. Circles are the locations of the data (for records with geographical coordinates), with colors indicating the number of records. The blue region refers to the Mediterranean climate area following Quézel & Médail^26^.

The geographical scope of the database is the Mediterranean Basin, defined as the land around the Mediterranean Sea with Mediterranean climate, including all Mediterranean islands. Given that the definition of Mediterranean climate is not strict (it has no sharp boundaries at the local scale), and that different Mediterranean species do not share their distribution limits, the border of our study area is also coarse – although as a general geographical reference we follow the limits of the Mediterranean climate by Quézel & Médail^26^ (Fig. 2). That is, some data come from locations outside of this geographical limit (Fig. 2) but these locations are close and share many species and relevant ecological processes (e.g. summer drought and frequent fires) with ecosystems in the core area of the Mediterranean climate.

We compiled measurements and observations on plant functional traits from scientific papers, books, reports, theses, and other types of publications for plants from the Mediterranean Basin. In some cases, we also gathered functional trait information from unpublished sources such as expert opinion. The compilation is based on the traits defined in BROT 1.0 (ref. 24), but also includes 30 additional traits; some of the original traits have been redefined in line with the current knowledge. The final 44 plant functional traits considered include 22 general vegetative traits, 15 regeneration traits, and 7 sexual reproductive traits (Table 9). These functional traits reflect the physiological, morphological, reproductive, phenological, and regenerative properties of plants, and determine the ecological role of each plant species in the ecosystem. Many of the functional traits included in the database are known to be important for the response of plants to global changes and disturbances^2,6,27,28^. For each trait, we provided a standardized definition, and in many cases, this definition includes both quantitative, semi-quantitative and qualitative information (see ‘Data Records’ section), which maximize gathering the available information. In some cases, the data is presented in a conditional form, that is, the data value includes some relevant extra information after a vertical bar (|, ASCII 124; i.e., *data*|*condition*; see examples in Table 5).

For some traits (eight traits, called ‘fixed’ traits in Table 9), only one value for each taxon was compiled; these traits are assumed to be constant across the study area. However, for most traits (36 traits, called ‘variable’ traits in Table 9) we compiled information from multiple sources and localities. This information may include different type (and quality) of data (e.g., quantitative or binary), but may also include different, sometimes even contradictory information. This variability in the data may reflect within-species variability, plasticity or poor knowledge, and thus it is an area for further research. The selection of fixed/variable attribute of the traits was based on the variability observed when gathering the data, and on the general ecological knowledge of the region.

The database only includes taxa (at least at the species level) native to the Mediterranean Basin. We have tried to avoid information obtained from different publications that were based on the same experiment (in many cases, different publications by the same authors). Functional trait data obtained from inadequate experimental procedures or data that did not fully fit the definition of a particular trait were excluded.

When available, we provided geographic information data (latitude, longitude, and altitude) for individual records in the database. In many cases, the reference from where the functional trait information was extracted did not include the coordinates, and thus we estimated them from the name of the locality (when possible). Because the studies vary from small plots to large regions, the coordinates of different record differ in the degree of accuracy in relation to the data. Thus we included the variable ‘Accuracy’ in the data to refer to the accuracy of the geographical location (see details in Table 8). Geographical information is provided for the variable traits only as fixed traits are assumed constant across the region.

Taxa names were homogenized following the European Science Foundation - European Documentation System (ESFEDS)^29^, which is largely based on Flora Europaea^30^. When a taxon name was missing in the ESFEDS database, or when some important taxonomic updates were available, generic taxonomical databases^31,32^, taxonomic databases for specific families^33,34^, or regional floras^35–37^ were used. Finally, all taxa names were checked for up-to-date synonymous names and spelling errors using the Taxonomic Name Resolution Service^38^. All BROT 2.0 full taxa names, including authority, are provided in the Taxa file. This file also includes the taxonomic family following the APG IV system^39^. In a separate file (the Synonymous file; Fig. 1), we include synonymous names for the taxa considered; this is not an exhaustive list and only includes some alternative names frequently used in the literature.

### Code availability

The four files of the database are in ‘CSV’ format and can be easily retrieved from Figshare (Data Citation 1) and uploaded to most statistical software, spreadsheets, or database management systems. The Sources, Taxa and Synonymous files have some special characters (e.g., accents on authors and authority names), and thus, for portability, UTF-8 encoding is used; the Data file uses plain text. Missing values are included as empty cells, and only occur in the Data file (in the last 5 columns; i.e., coordinates and comments; Table 1) and Taxa file (in the last two columns; Table 2).

As an example, we provide below the code to import the data into R and to perform some simple tasks; this code was tested in R (ver. 3.4) under Windows (ver. 7 and 10) and Linux (Ubuntu 16.04) and should work in any of the main computer configurations. Importing the files into a spreadsheet is easy, especially for LibreOffice and OpenOffice; users just need to select 'Unicode UTF-8’ and fields separated by ‘comma’ when opening csv files. Microsoft Excel users may need to first rename the four *.csv files as *.txt, open them via the Import Text Wizard, and then select 'Unicode UTF-8’ with fields separated by ‘comma’.

Reading the BROT 2.0 files:

brot <- read.csv("BROT2_dat.csv", row.names=1, stringsAsFactors=F)

brot.sou <- read.csv("BROT2_sou.csv", row.names=1, stringsAsFactors=F, encoding="UTF-8")

brot.tax <- read.csv("BROT2_tax.csv", row.names=1, stringsAsFactors=F, encoding="UTF-8")

brot.syn <- read.csv("BROT2_syn.csv", stringsAsFactors=F, encoding="UTF-8")

Quantifying the variability (quantiles) of SLA values:

quantile(as.numeric(brot$Data[brot$Trait=="SLA"]))

Plotting the number of species by growth forms:

barplot(sort(table(brot$Data[brot$Trait=="GrowthForm"])), las=2)

Does *Daphne gnidium* resprout after fire?

brot[brot$Taxon=="Daphne gnidium" & brot$Trait=="RespFire", 1:8]

And what about *Quercus* species? Do they resprout after fire?

with(brot[grep("Quercus", brot$Taxon),], table(Data[Trait=="RespFire" & DataType %in% c("boolean", "semi-quantitative")]))

How many species show their germination stimulated by smoke?

length(unique(brot$Taxon[brot$Data=="stimulation|smk"]))

Summarize the role of the different fire-related chemical cues on plant germination:

with(data.frame(matrix(unlist(strsplit(brot$Data[brot$Trait=="ChemCues"], "|", fixed=T)), ncol=2, byrow=T)), table(X1, X2))

List the references that provide information on lignotubers:

sort(brot.sou[unique(brot$Source[brot$Data=="lignotuber"]), ])

Add families to all records in the main data file:

brot$Family <- brot.tax[as.character(brot$TaxonID), "Family"]

List all synonymous used in BROT (two ways with different output formats):

merge(brot.syn, brot.tax[,-1], by.x="TaxonID", by.y=0)[,-1]

apply(merge(brot.syn, brot.tax[,-1], by.x="TaxonID", by.y=0)[,-1], 1, function(x) paste(x[1], paste(na.omit(x[-1]), collapse=" "), sep="=" ))

## Data Records

The data compiled is available in a single dataset (Data Citation 1) composed of four ASCII text files, all in CSV format with quoted fields: the ‘Data’ file (BROT2_dat.csv) is the main file; the ‘Taxa’ file includes full taxa names (BROT2_tax.csv), and the ‘Sources’ file includes full references (BROT2_sou.csv). An additional ‘Synonymous’ file (BROT2_syn.csv) includes synonymous names for some of the taxa in the ‘Taxa’ file.

**Figure 3:**
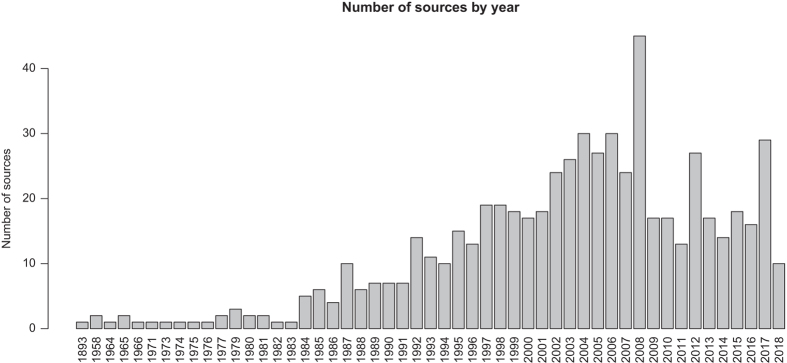
Number of sources included in the database by year of publication.

**Figure 4:**
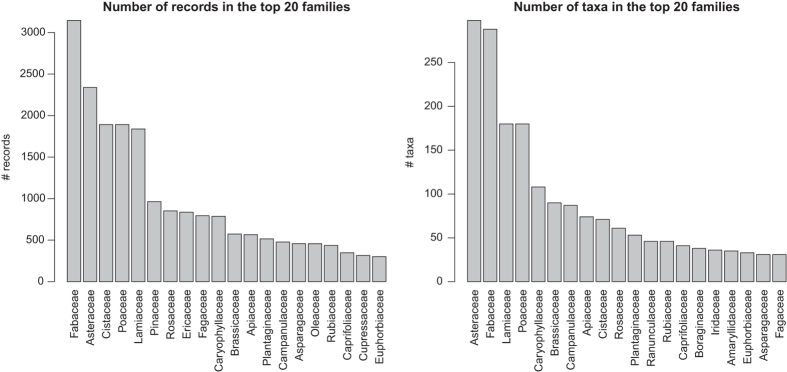
Number of records (left) and number of taxa (right) for the best represented (top 20) families in the database.

**Figure 5:**
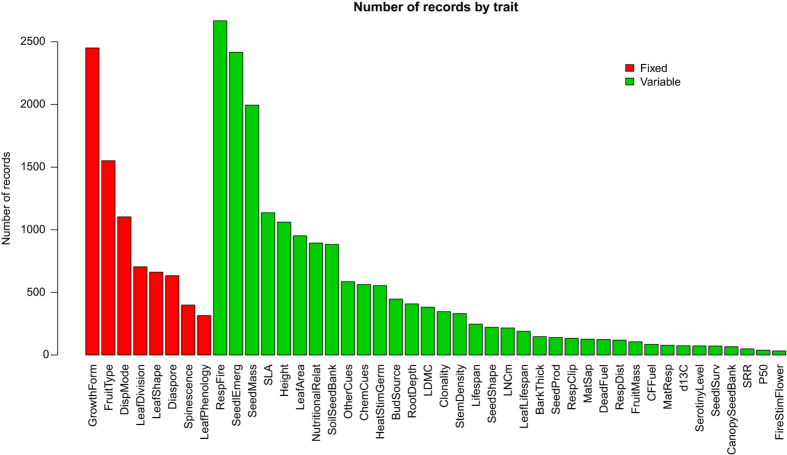
Number of records in the database for each trait. It includes both fixed (in red) and variable traits (in green). Trait codes as in Table 9.

In total, we compiled functional trait data from 624 sources, of which 448 are articles published in peer-reviewed journals between 1893 and early 2018 (Fig. 3). The database includes 25,764 records for 2,457 taxa belonging to 2,265 species, 704 genera, and 119 families throughout the Mediterranean Basin. Asteraceae, Fabaceae, Lamiaceae, Poaceae, and Cistaceae are the best represented families in BROT 2.0, with the specific ranking depending on whether we consider the number of records or the number of species (Fig. 4). The top traits in number of records were RespFire (2,668), GrowthForm (2,451), SeedlEmerg (2,417), SeedMass (1,995), and FruitType (1,551 records) (Fig. 5). Data records are geographically distributed throughout the Mediterranean Basin, but some parts of the basin (e.g. the southern rim) are poorly represented (Fig. 2), reflecting the lower number of available studies in this area. Most of the data records (12,132) come from 379 studies conducted in the Iberian Peninsula, following by Greece (2,366 records from 59 sources), Anatolia (1,303 records from 45 sources), Mediterranean France (1,302 records from 38 sources), and Italy (761 records from 27 sources). Considering the numbers of data records, taxa, and sources summarized above, BROT 2.0 represents a significant improvement (both quantitatively and qualitatively) over BROT 1.0 (ref. 24) (Table 10).

Definitions, categories, and units of functional traits included in the database are given below, with the trait short names (as in the Data file, Table 1) and whether it is a fixed or variable trait (F, V) in brackets. Traits are ordered following Table 9, that is, first are vegetative traits (1 to 22), then sexual reproductive traits (23 to 29), and finally regeneration traits (30 to 44). Note that the trait short names (in brackets) and all categories (in bold) are written as used in the database (Data file), including the letter case.

### 1 Growth form (GrowthForm, F)

Morphology of the whole plant related with its size (for non-disturbed individuals). Categories are:

- **tree:** very tall woody plant, frequently with one main, primary stem and the green canopy rarely reaching the ground.
- **large shrub:** Large shrub or small tree. Tall woody plant that under optimal conditions may reach arborescence structure.
- **shrub:** woody plants (typically less than 1.5 m), frequently with several shoots growing from the soil level and/or the green canopy reaching the ground.
- **liana:** woody (or slightly ligneous at the base) climber.
- **subshrub:** dwarf woody plant or chamaephytes (typically less than 50 cm), including suffruticose (suffrutescent) plants.
- **perennial forb:** perennial broad-leaved herbaceous plant.
- **perennial graminoid:** perennial grass-like plant.
- **annual forb:** annual broad-leaved herbaceous plant.
- **annual graminoid:** annual grass-like plant.
- **variable forb:** annual, biennial or short-perennial forb.
- **variable graminoid:** annual, biennial or short-perennial grass.
- **geophyte:** herbs that persist during the unfavorable period as bulbs, rhizomes or other subterranean storage organs.
- **epiphyte:** plants growing on other plants for physical support.

### 2 Leaf division degree (LeafDivision, F)

Division degree of leaves (or phyllodes). For species with different stem and basal leaves, it refers to stem leaves. The categories are:

- **simple:** simple leaves.
- **compound:** compound or divided leaves.

### 3 Average leaf lifespan (LeafLifespan, V)

Average time period (months) during which an individual leaf (or phyllodes) of a woody species is alive and physiologically active^40^ (= leaf longevity). For woody species only.

### 4 Leaf phenology (LeafPhenology, F)

Phenology of leaves (or phyllodes). For woody species only. The categories are:

- **evergreen:** plant that maintains green leaves all year.
- **winter deciduous:** plant that drops all its leaves during the winter.
- **winter semi-deciduous:** plant that drops part of its leaves during the winter, maintaining some brownish leaves in the crown.
- **drought semi-deciduous:** plant that drops part of its leaves during the dry period (excluding species that drop leaves only in very extreme droughts).

### 5 Basic leaf shape (LeafShape, F)

Shape of leaves, phyllodes or leaflets for compound leaves. For species with different stem and basal leaves, it refers to stem leaves. The categories are:

- **none:** without leaves or any functional analogue organ. If leaves are modified as spines, then LeafShape=spines (see below).
- **broad:** plant with broad leaves (for compound leaves, this refers to leaflets).
- **needle-like:** plant with needle-like leaves.
- **linear:** plant with linear leaves (for compound leaves, this refers to leaflets).
- **scale-like:** plant with scale-like leaves.
- **spines:** leaves are modified as spines (which is different from having spines or thorns in the branches).
- **succulent:** plant with succulent (water-stored, thick and fleshy) leaves.

### 6 Average leaf area (LeafArea, V)

Average one-sided projected surface area (mm^2^) of an individual leaf (or phyllodes)^40^. For compound leaves, leaflets area×leaflets number. For species with different stem and basal leaves, it refers to stem leaves. Alternatively, one of the following categories:

- **very small:** small needle-like, scale-like, and linear leaves (typically less than 25 mm^2^).
- **small:** large linear leaves or small broad leaves (typically 25–225 mm^2^).
- **medium:** moderate broad leaves or divided leaves with moderate leaflets (typically 225–2025 mm^2^).
- **large:** large broad leaves, or divided leaves with numerous and large broad leaflets (typically 2025–4550 mm^2^).
- **very large:** very large broad leaves, usually divided (typically more than 4550 mm^2^).

### 7 Mass-based leaf nitrogen content (LNCm, V)

Leaf nitrogen content (mass-based) (mg g^−1^), that is, the ratio of the quantity of nitrogen in the leaf per respective unit dry mass^40,41^.

### 8 Average specific leaf area (SLA, V)

Average one-sided area of the fresh leaf (or phyllodes) divided by its oven-dry mass^40,42^, excluding petiole and/or rachis; for mature plants only. Units are mm^2^ mg^−1^ (note that mm^2^ mg^−1^×10=cm^2^ g^−1^).

### 9 Average bark thickness (BarkThick, V)

Average bark thickness (mm) of the main stem at breast height for trees; if available, it should include the diameter (cm) at the height that the bark was measured (as quantitative conditional, i.e., BT|Diam). For woody plants only. Note that in many cases both BT and Diam are mean values of a population (not for an individual tree). Alternatively, one of the following categories:

- **thin:** <= 2 mm.
- **moderate:** 2–15 mm.
- **thick:** >15 mm.

### 10 Average height (Height, V)

Maximum height (m), excluding extremes and reproductive shoots.

### 11 Stem specific density (StemDensity, V)

Oven-dry mass divided by the fresh volume of a section of the main stem^40^ (excluding bark for woody species; i.e., wood density) (g cm^-3^).

### 12 Coarse:fine fuel (CFFuel, V)

Coarse to fine fuel biomass ratio (i.e., >=6 mm diameter and<6 mm diameter, respectively), including live and dead material. Alternatively, one of the following categories:

- **low:** without coarse fuel; all fuel is fine.
- **moderate:** abundant fine fuel and low coarse fuel.
- **high:** abundant fine fuel and abundant coarse fuel.
- **very high:** scarce fine fuel and abundant coarse fuel.

### 13 Dead fine fuel (DealFuel, V)

Standing fine (< 6 mm diameter) dead biomass (including twigs, leaves, inflorescences, bark) attached to the plant during the dry season expressed in proportion of the aboveground biomass (for non-senescent individuals) (%). Alternatively, one of the following categories:

- **low:** <=5%
- **medium:** 5-20%
- **high:** >=20%

### 14 Leaf dry matter content (LDMC, V)

Dry matter content of leaves (mg g^−1^), that is, the ratio of the dry mass of a leaf to its water saturated fresh mass^40,41^.

### 15 Clonality (Clonality, V)

Ability to colonize the space through vegetative reproduction. Categories considered are:

- **rhizomes:** non-swollen belowground horizontal stem that grows near the soil surface with the ability to produce roots and stems. Rhizomes s.l., i.e., including woody and non-woody rhizomes.
- **roots:** roots normally growing close to the soil surface.
- **rhizomes or roots:** rhizomes and/or roots (not differentiated).
- **storage organs:** non-woody storage organs, normally modified stems (bulbs, corms or stem tubers) or roots (root tubers).
- **stolons:** aboveground horizontal stems.
- **yes:** clonal plant with unknown system.
- **no:** without clonality ability.

### 16 Integrated water-use efficiency (d13C, V)

Delta 13C (δ13C) isotope ratio^40^.

### 17 Lifespan (Lifespan, V)

Average maximum age (=longevity) for perennials (year). Alternatively, one of the following categories:

- **very short:** <=2 yr
- **short:** 2–5 yr
- **medium:** 5–25 yr
- **long:** 25–150 yr
- **very long:** >150 yr

### 18 Nutritional relationships (NutritionalRelat, V)

Long-term relationship with other species that are implicated in nutrient and/or energy uptake. Categories are:

- **ectomycorrhizal symbiosis:** plant with a mutualistic relationship with ectomycorrhizal fungi.
- **endomycorrhizal symbiosis:** plants with a mutualistic relationship with endomycorrhizal fungi.
- **nodule bacteria symbiosis:** plant with a mutualistic relationship with nitrogen-fixing bacteria.
- **hemiparasite:** photosynthetic plant (hemiparasite) that parasitize another photosynthetic plant (host).
- **parasite:** non-photosynthetic plant that parasitize either a photosynthetic plant (i.e. holoparasites) or a fungus (i.e., myco-heterotrophous).
- **carnivorous:** plant acquiring nutrients by capturing animals or protozoa.
- **others:** plants with other types of nutritional relationships, including a combination of several types of symbiosis (e.g. endo- and ectomycorrhizas).
- **no:** plant without obvious nutritional relationships.

### 19 Resistance to xylem cavitation (P50, V)

Xylem resistance to drought-induced cavitation (in MPa)^5^.

### 20 Maximum rooting depth (RootDepth, V)

Maximum depth of the roots (m).

### 21 Spinescence (Spinescence, F)

Presence/absence of spines, thorns, prickles and/or spiny leaves in vegetative organs. The categories are:

- **yes**
- **no**

### 22 Shoot:root ratio (SRR, V)

Shoot to root dry mass ratio in saplings (<=3 years old), excluding those from plantations. Alternatively, one of the following categories:

- **low:**<1.5
- **high:** >=1.5

### 23 Dispersal mode (DispMode, F)

The vector(s) used for dispersal (of seeds or any other dispersal unit for sexual reproduction). One or several types, e.g.: GW means that both Gravity and Wind are important); if several, typically the first is the most important. Notation for the different dispersal vectors are:

- **G:** autochory, by Gravity (=unassisted dispersal).
- **W:** anemochory, by Wind (with wind dispersal adaptations).
- **H:** Hydrochory, by water.
- **B:** Ballistichory, by launching (=ballochory).
- **M:** Myrmecochory, by ants.
- **N:** eNdozoochory, internal animal transport.
- **P:** ePizoochory, external animal transport (=exozoochory).
- **O:** hOarding, scatter and hoarding diaspores by animals (others than ants).
- **Z:** Zoochory, dispersal mediated by animals (unknown transport system).

### 24 Diaspore (Diaspore, F)

Dispersal unit for sexual reproduction. The categories are:

- **seed:** including some single-seeded fruits such as achenes or caryopsis.
- **fruit:** single or aggregated fruit.
- **spore**

### 25 Fruit dry mass (FruitMass, V)

Average dry mass (mg) of single or aggregated fruits (excluding some single-seeded fruits such as achenes or caryopsis, that are typically considered as "seeds"). Alternatively, one of the following categories:

- **very light:**<3 mg
- **light:** 3–30 mg
- **medium:** 30–300 mg
- **heavy:** 300–3000 mg
- **very heavy:** >3000 mg

### 26 Fruit type (FruitType, F)

Type of the fruit. Including single or aggregated fruits (dispersal unit). Categories are:

- **dry:** dry fruit.
- **fleshy:** fleshy fruit, including fruit in which the fleshy part is the floral cup (hypanthium) (e.g. *Rosa*).

### 27 Seed dry mass (SeedMass, V)

Average dry mass of seeds (including some single-seeded fruits such as achenes or caryopsis) (mg). Alternatively, one of the following categories:

- **very light:**<3 mg
- **light:** >=3 and<30 mg
- **medium:** >=30 and<300 mg
- **heavy:** >=300 mg

### 28 Annual seed production (SeedProd, V)

Average number of seeds per plant produced every year (# seeds). Alternatively, one of the following categories:

- **rarely:** rarely, if ever, produces seeds in the study area.
- **few:** <=50 seeds.
- **medium:** 50–500 seeds.
- **many:** > 500 seeds.

### 29 Basic seed shape (SeedShape, V)

Ratio between the two maximum diameters: first maximum divided by the second maximum, excluding structures attached to the seed coat as wings or pappus. Alternatively, one of the following categories:

- **regular:** close to 1 (spherical or lens-shaped seeds).
- **irregular:** far to 1 (elongated seeds).

### 30 Bud source (BudSource, V)

Location of the bud bank for resprouting species^43^. The categories are:

- **epicormic buds:** stem buds (protected by the bark).
- **apex:** buds in the stem apex protected from fire by leaf bases.
- **root crown:** transition point between stem and root.
- **lignotuber:** woody swelling below or just above the soil, ontogenetically programmed (i.e., inherited character). Based on embryological and/or anatomical features.
- **thickened root-crown:** woody swelling below or just above the soil non-ontogenetically programmed (e.g. stem coalescence). Thickened root crown.
- **burl:** woody swelling below or just above the soil with the unspecified origin (no distinction between lignotuber and burl is reported).
- **rhizomes:** belowground horizontal stem (non-swollen).
- **woody rhizomes:** woody belowground horizontal stem.
- **non-woody rhizomes:** non-woody belowground horizontal stem.
- **roots**
- **rhizomes or roots:** rhizomes and/or roots (unspecified).
- **storage organs:** non-woody storage organs, i.e., modified stems (bulbs, corms or stem tubers) or roots (root tubers).
- **others:** other bud sources, including those not clearly specified (e.g. stump).

### 31 Fire-stimulated flowering (FireStimFlower, V)

Fire-stimulated flowering in post-fire resprouters. The categories are:

- **no:** lower or similar flowering post-fire than in unburned vegetation.
- **yes:** higher flowering post-fire than in unburned vegetation (includes facultative and obligate post-fire flowering); these are mostly geophytes.

### 32 Resprouting capacity after clipping (RespClip, V)

Resprouting capacity one year after clipping 100% of the plant as average proportion of adult plants that resprout (percentage). Not reported for annual plants (which can be assumed to be RespClip=no). Alternatively, one of the following categories:

- **no:** without resprouting capacity.
- **low:** few individuals resprouting and/or weak resprouts.
- **high:** most individuals resprouting and/or vigorous resprouts.
- **yes:** with resprouting capacity (not quantified).
- **variable:** very high variability observed in the sampling area.

### 33 Resprouting capacity after disturbances (RespDist, V)

Resprouting capacity after undefined disturbances (average proportion of adult plants that resprout as percentage). Not reported for annual plants (which can be assumed to be RespDist=no). Alternatively, one of the following categories:

- **no:** without resprouting capacity.
- **low:** few individuals resprouting and/or weak sprouts.
- **high:** most individuals resprouting and/or vigorous sprouts.
- **yes:** with resprouting capacity (not quantified).
- **variable:** very high variability observed in the sampling area.

### 34 Resprouting capacity after fire (RespFire, V)

Resprouting capacity after one year when most of the plant has been scorched (average proportion of adult plants that resprout as percentage). Not reported for annual plants (which can be assumed to be RespFire=no). Alternatively, one of the following categories:

- **no:** without resprouting capacity.
- **low:** few individuals resprouting and/or weak sprouts.
- **high:** most individuals resprouting and/or vigorous sprouts.
- **yes:** with resprouting capacity (not quantified).
- **variable:** very high variability observed in the sampling area.

### 35 Chemical germination cues (ChemCues, V)

Germinative response to smoke (smk), ash (ash), charcoal (cha), nitrogenous compounds like KNO_3_ (NC1), NaNO_2_ (NC2), NH_4_Cl (NC3), NH_4_HOC_3_ (NC4), NH_4_NO_3_ (NC5), or response to karrikins (KAR), cyanohydrins (CYN, including several cyanides, mandelonitrile, and glyceronitrile). The response in indicated before the vertical bar, and the chemical cue tested, after the vertical bar (e.g. stimulation|smk). The categories are:

- **stimulation|###:** germination of the treated seeds higher than the control.
- **unaffected|###:** germination of the treated seeds equal to the control.
- **inhibition|###:** germination of the treated seeds lower than the control.

### 36 Heat-stimulated germination (HeatStimGerm, V)

The highest intensity in heat treatments (i.e., seed exposition to dry heat >=50 °C) that produce higher germination than the control (i.e., stimulated germination). Heat intensity defined as: Low (L:<100 °C during <=5 min.), Moderate (M:<100 °C during >5 min. or >=100 °C during <= 5 min.), High (H: >=100 °C during >5 min.) or unknown (unk). Note that in many cases, post-treatment seed viability is not considered or not specified. The heat intensities tested for each experiment are indicated after the vertical bar (*category*|LMH), with an underscore when the corresponding heat intensity is not tested (e.g., *category*|L_H). The categories are:

- **yes|unk:** stimulated germination is produced after unspecified heat intensity exposition.
- **high|###:** stimulated germination after exposition to High-intensity treatments (### refers to L, M and H respectively).
- **moderate|###:** the highest heat intensity that produces stimulated germination is Moderate (### refers to L, M and H respectively).
- **low|###:** the highest heat intensity that produces stimulated germination is Low (### refers to L, M and H respectively).
- **unaffected|###:** germination is not stimulated after any heat intensity tested and at least one of the treatments does not affect seed germination (### refers to L, M and H respectively; unk if unknown).
- **inhibition|###:** inhibited germination (i.e., lower germination than control) in all heat treatments tested (### refers to L, M and H respectively; unk if unknown).

### 37 Other germination cues (OtherCues, V)

Germination response to exposition to boiling water (blw), mechanical scarification (mec), summer temperature (tsu), temperature fluctuation (tfu), light (lgt). The response is indicated before the bar and the cue tested is indicated after the vertical bar (*category*|*cue*, e.g., stimulation|blw). Categories are:

- **stimulation|###:** germination of the treated seeds higher than the control.
- **unaffected|###:** germination of the treated seeds equal to the control.
- **inhibition|###:** germination of the treated seeds lower than the control.

### 38 Canopy seed bank longevity (CanopySeedBank, V)

Presence and longevity of the stored seeds in the canopy. The categories are:

- **yes:** seeds stored in the canopy is present (no information on longevity)
- **no:** no stored seeds in the canopy.
- **short:** 2 or 3 years
- **mid:** 4–10 yr
- **long:** >10 yr

### 39 Serotiny level (SerotinyLevel, V)

Average proportion of serotinous (closed) cones in the canopy (%)^44^.

### 40 Soil seed bank longevity (SoilSeedBank, V)

Period that seeds remain viable in the soil seed bank inferred from: soil and vegetation comparisons (veg), experimental seed burial (bur), seed dormancy (dor) or unknown methods (unk). The method used is indicated after the vertical bar (*response*|*method*, e.g., persistent|veg). The categories are:

- **transient|###:** no soil seed bank; seeds germinate in the first favorable season after dispersal. Normally seed bank longevity <=1 yr (no persistent seed bank).
- **persistent|###:** seeds do not germinate in the first favorable season after dispersal. Normally seed bank longevity >1 yr (could be longer but it is unknown).
- **short-persistent|###:** >1 and <=5 yr
- **at least short-persistent|###:** at least>1 and <=5 yr (could be longer but it is unknown).
- **mid-persistent|###:** at least>5 yr (could be longer but it is unknown)
- **long-persistent|###:** at least>15 yr
- **very long-persistent|###:** >=30 yr

### 41 Age at maturity of resprouts (MatResp, V)

Average age of resprouts at the first successful reproduction (yr), i.e. when most of the resprouted plants produce the first seed crop. Alternatively, one of the following categories:

- **early:**<5 yr
- **medium:** 5–10 yr
- **late:** >10 yr

### 42 Age at maturity of saplings (MatSap, V)

Average age of saplings at the first successful reproduction (yr), i.e. when most of the saplings produce the first seed crop, excluding saplings from plantations. Alternatively, one of the following categories:

- **early:**<5 yr
- **medium:** 5–10 yr
- **late:** >10 yr

### 43 Post-fire seedling emergence (SeedlEmerg, V)

Average density of seedlings per pre-fire mature individuals emerged during the first year after fire. This is a ratio (number of seedlings / number of mature plants), or alternatively, one of the following categories:

- **no:** no post-fire seedlings emergence.
- **low:** number of seedlings lower than the number of pre-fire mature individuals.
- **high:** number of seedlings higher than the number of pre-fire mature individuals.
- **yes:** post-fire seedling emergence observed (quantitative data not available).
- **variable:** high variability observed between populations or sampled areas.

### 44 Post-fire seedling survival (SeedlSurv, V)

Proportion of seedlings surviving the first dry season after fire (%). Alternatively, one of the following categories:

- **no:** no post-fire seedlings survival.
- **low:** survival<25%
- **high:** survival >=25%

## Technical Validation

Most of the records included in the database (79%) are based on published material in peer-reviewed scientific journals (46%), books (27%), or theses (5%), and thus most data should be accurate. In addition, each specific data value has the original references, and so users can evaluate the validity and accuracy of the original source. The data has been checked for possible redundancies and errors, and some published data were excluded from the final database; data considered doubtful (e.g., very extreme values, or data that did not fully fit the definition of the trait, or that were obtained with a questionable sampling) were not included. Also note that for each species and trait, the BROT 2.0 database includes different values from different sources, and so users can make their own decisions on data usage; for example, using the mean trait value, or excluding extreme values. This feature of BROT 2.0 database is especially important because it provides information on trait variability.

The spatial scope of the database is the Mediterranean Basin (Fig. 2). The differences in the number of records among sub-regions of the Basin (for example, the north and the south of the Basin, Fig. 2) reflect the spatial heterogeneity of knowledge across the Basin.

The integrity of the files can be verified by the following md5 checksums:

BROT2_dat.csv "797222960172b5ca587173f061a5f629"

BROT2_sou.csv "302c8f6bbaa2f1a49c95f6706941941c"

BROT2_tax.csv "758a4c23a30b080333face4c6689c47b"

BROT2_syn.csv "c756db95e433283b6c2bdca51a11a5e3"

## Usage Notes

To properly use the data, it is important to consider the following fields in the Data file: ‘Method’, ‘Accuracy’, and ‘DataType’. The first provides information on the origin of the data, and is related to quality; data with Method= ‘measure’ is likely to be of the highest quality (Table 6). ‘Accuracy’ provides an indicator of the accuracy of the geographical location (Table 8). Users can use these fields to select the data that best suits their requirement, or to weight their analysis. The ‘DataType’ column is also informative, as different types of data (quantitative, semi-quantitative, etc.) are linked to the quality and accuracy of the data (Table 5). Because quantitative data can be converted to qualitative or semi-quantitative (following the trait definitions given in the section ‘Data Records’), users can choose between using more data of low quality or less data of high quality.

Note that there may be variability in a given trait, and the more information (more records from different sources), the more certain is the value of the trait for a given species. For instance, resprouting is a key trait in fire-prone ecosystems, and for some (few) species, we may find records for both ‘yes’ and ‘no’ in the ability to resprout. This may be due to variability, but it could also be due to a poor sampling. The number of records, together with the field ‘Methods’, may provide an estimation of the level of confidence in the information. Moreover, given that all records are referenced, it is always possible to go to the original reference to search for additional information.

Note also that some traits are poorly known and prone to mistakes. For instance, the type of underground resprouting (trait: ‘BudSource’) is not always easy to observe, and many researchers, especially in the past, tended to confuse structures like ‘lignotubers’ with any other underground resprouting organ^43,45^. When the mistake was clear from the reference, it was not included in the database, but in many instances, it is impossible to know, as variability exists among populations and geographical locations.

## Additional information

**How to cite this article**: Tavşanoğlu, Ç. & Pausas, J. G. A functional trait database for Mediterranean Basin plants. *Sci. Data* 5:180135 doi: 10.1038/sdata.2018.135 (2018).

**Publisher’s note**: Springer Nature remains neutral with regard to jurisdictional claims in published maps and institutional affiliations.

## Supplementary Material



## Figures and Tables

**Table 1:** Column description of the Data file.

Column name	Definition
ID	Unique identifier.
TaxonID	Unique taxa identifier; the ID in the Taxa file (Table 2, Fig. 1).
Taxon	Taxa name without author names; see the Taxa file for authorities (see Table 2).
Trait	Name of the functional traits considered (list and definitions are given in Table 9 and in ‘Data Records’ section).
Data	The actual data. The categories for each functional trait are described in ‘Data Records’ section; words are not capitalized, all text is in lower case except single-letter codes (e.g. DispMode).
Units	Units for quantitative data; number of classes (in squatted brackets) for categorical and semi quantitative data.
DataType	Type of data as defined in Table 5. Note that the database includes different types of data, even for a given trait.
Method	General description of the method for gathering the information; it is related to the accuracy of the data (see Table 6).
SourceID	Unique identifier for the data source (Fig. 1) from which data have been obtained. Complete references are listed in the Source file (see Table 3).
Region	The region of the Mediterranean Basin where the observation or experiment was performed or from where the seeds were collected (see Table 7).
Lat	Latitude (in decimal degree) of the study site. This field can be empty.
Long	Longitude (in decimal degree) of the study site. This field can be empty.
Alt	Altitude (in m) of the study site. This field can be empty.
Accuracy	The accuracy of the geographical coordinates. The definitions of the study sites range from small plots to large regions, and thus the given geographical coordinates may vary in their degree of accuracy in relation to the data (see Table 8). This field can be empty.
Comments	Some data has a brief comment or clarification provided by the author (indicated as ‘f.a.’) or by the data compiler (indicated as ‘f.c.’). This field can be empty.

**Table 2:** Column description of the Taxa file.

Column name	Definition
ID	Unique identifier of the taxa.
Family	APG IV family^39^
Genus	Genus, the first part of the species binomial name
Species	Specific epithet, i.e., the second part of the species binomial name
Authority1	Authority for the species binomial name
Infraname	Two words separated by a space; the first is ‘spp.’ or ‘var.’ referring to subspecies or variety, respectively, the second is the name of the subspecies or variety. This field can be empty.
Authority2	Authority for the name of the subspecies or variety. This field can be empty.

**Table 3:** Column description of the Sources file.

Column name	Definition
ID	Unique identifier.
FullSource	Full reference. Note that the sources include published articles, gray literature, and personal communications; in the latter, the email of the data provider and a brief description of the study area are also included. Sources follow the APA Style^46^ with some minor modifications.

**Table 4:** Column description of the Synonymous file.

Column name	Definition
TaxonID	Taxa identifier that links with the Taxa file (ID) and the Data file (TaxonID); see Fig. 1. This is not unique in this file, as several synonymous for the same taxa are possible.
SynName	Full taxa name, with authorities.

**Table 5:** Descriptions of types of data used in the Data file (the ‘DataType’ column).

DataType	Definition
Quantitative	A number (integer or floating)
Semi-quantitative	Ordered qualitative variable (e.g., low, medium, high)
Range	Two quantitative values, indicating the range observed (e.g., 20–100)
Categorical	Non-ordered qualitative variable (e.g, dry, fleshy)
Boolean	A value indicating a logical expression (yes or no)
Conditional	The above types may also, in turn, be conditional (quantitative conditional, semi-quantitative conditional, etc.) and indicate that the data entry has a value plus some additional information separated by a vertical bar (|, ASCII 124). This additional information is different in each trait, and it is defined in the corresponding traits (see ‘Data Records’ section). Here are three examples: HeatStimGerm=“low|L_H”: germination stimulated by low heat intensities in an experiment where only Low and High heat intensities were tested (as defined in ‘Data Records’ section). That is, it is unknown whether it would be stimulated by a moderate heat intensity treatment; it was not stimulated by a high heat shock. ChemCues=“stimulation|smk”: germination stimulated by smoke treatments (i.e., germination after smoke treatment was significantly higher than the germination in control conditions). SoilSeedBank=“persistent|bur”: plant that has persistent soil seed bank according to the evidence come from a seed burial experiment.

**Table 6:** Descriptions of general methods of published or unpublished data sources (the ‘Method’ column in the Data file).

Method	Definition
Measure	Published or unpublished data obtained from an experimental design in which the data is, at least, one of the objectives of the study.
Experience	Published or unpublished data from visual (rough) estimation or personal experience.
Compilation	Published data compiled from different sources (including experience, published data,...).
General reference	Data published and obtained from a general publication such as a regional flora.

**Table 7:** Descriptions of regions used in the Data file (the ‘Region’ column).

Region	Definition
W	North West: Iberian Peninsula, south of France and Balearic Islands.
C	North-Central: Italian Peninsula and surrounding islands (Sicily, Sardinia, Corsica).
E	North East: from Trieste to Istanbul, that is, Croatia, Albania, the Former Yugoslav Republic of Macedonia, Greece, and surrounding islands.
M	Mediterranean Middle East (Asia): From Istanbul to the Sinai Peninsula. That is Anatolian Peninsula, western Syria, Lebanon, Palestine, Israel, and Cyprus.
S	The Southern rim of the Mediterranean Sea (North Africa), that is, Morocco, Algeria, Tunisia, Libya, and Egypt.
U	Unknown, or unclear in the original source, or from more than two of the above regions.

**Table 8:** Description of accuracy categories for geographic coordinates used in the Data File (the ‘Accuracy’ column).

Accuracy	Definition
High	Data refer to a region of<5 km^2^ in size.
Mod	Data refer to a region between 5 and 500 km^2^ in size.
Low	Data refer to a region between 500 and 50 000 km^2^ in size.
VLow	Data refer to a region >50 000 km^2^ in size.

**Table 9:** List of functional traits included in the database.

No	Trait	Trait full name	Data types	Trait type
***General vegetative traits***				
1	GrowthForm	Growth form	categorical	F
2	LeafDivision	Leaf division degree	categorical	F
3	LeafLifespan	Average leaf lifespan	quantitative	V
4	LeafPhenology	Leaf phenology	categorical	F
5	LeafShape	Basic leaf shape	categorical	F
6	LeafArea	Average leaf area	quantitative or semi-quantitative	V
7	LNCm	Mass-based leaf nitrogen content	quantitative	V
8	SLA	Average specific leaf area	quantitative	V
9	BarkThick	Average bark thickness	quantitative, quantitative conditional, or semi-quantitative	V
10	Height	Average height	quantitative	V
11	StemDensity	Stem specific density	quantitative	V
12	CFFuel	Coarse:fine fuel	quantitative or semi-quantitative	V
13	DeadFuel	Dead fine fuel	quantitative or semi-quantitative	V
14	LDMC	Leaf dry matter content	quantitative	V
15	Clonality	Clonality	categorical or Boolean	V
16	d13C	Integrated water-use efficiency	quantitative	V
17	Lifespan	Lifespan	quantitative or semi-quantitative	V
18	NutritionalRelat	Nutritional relationships	categorical	V
19	P50	Resistance to xylem cavitation	quantitative	V
20	RootDepth	Maximum rooting depth	quantitative	V
21	Spinescence	Spinescence	Boolean	F
22	SRR	Shoot:root ratio	quantitative or semi-quantitative	V
***Sexual reproductive traits***				
23	DispMode	Dispersal mode	categorical	F
24	Diaspore	Diaspore	categorical	F
25	FruitMass	Fruit dry mass	quantitative or semi-quantitative	V
26	FruitType	Fruit type	categorical	F
27	SeedMass	Seed dry mass	quantitative or semi-quantitative	V
28	SeedProd	Annual seed production	quantitative or semi-quantitative	V
29	SeedShape	Basic seed shape	quantitative or semi-quantitative	V
***Regeneration traits***				
30	BudSource	Bud source	categorical	V
31	FireStimFlower	Fire-stimulated flowering	semi-quantitative or Boolean	V
32	RespClip	Resprouting capacity after clipping	quantitative, semi-quantitative, categorical or Boolean	V
33	RespDist	Resprouting capacity after disturbances	quantitative, semi-quantitative, categorical or Boolean	V
34	RespFire	Resprouting capacity after Fire	quantitative, semi-quantitative, categorical or Boolean	V
35	ChemCues	Chemical germination cues	semi-quantitative conditional	V
36	HeatStimGerm	Heat-stimulated germination	semi-quantitative conditional or Boolean conditional	V
37	OtherCues	Other germination cues	semi-quantitative conditional	V
38	CanopySeedBank	Canopy seed bank longevity	semi-quantitative or Boolean	V
39	SerotinyLevel	Serotiny level	quantitative or quantitative range	V
40	SoilSeedBank	Soil seed bank longevity	semi-quantitative conditional	V
41	MatResp	Age at maturity of resprouts	quantitative or semi-quantitative	V
42	MatSap	Age at maturity of saplings	quantitative or semi-quantitative	V
43	SeedlEmerg	Post-fire seedling emergence	quantitative, semi-quantitative, categorical or Boolean	V
44	SeedlSurv	Post-fire seedling survival	quantitative or semi-quantitative	V
‘Trait’ refers to the short name of functional trait as used in the database (Data file, Table 1), ‘Trait full name’ is the full name of functional traits (see also ‘Data Records’ section), ‘Data types’ shows the types of data used in each functional trait (Table 5), and ‘Trait type’ shows whether a functional trait is fixed (F, i.e. one record for each species) or variable (V, any species may include several records reflecting variability of the trait).				

**Table 10:** Main differences between BROT 1.0 and BROT 2.0.

Feature	BROT 1.0	BROT 2.0
**# of traits**	14	44
# of general vegetative traits	1	22
# of sexual reproductive traits	1	7
# of regeneration traits	12	15
**# of taxa**	952	2,457
# of species	859	2,265
# of genera	384	704
# of families	79	119
**# of records**	8,263	25,764
with geographical coordinates	0	15,574
**# of sources**	301	624
